# Possible Modulation of the Anexiogenic Effects of *Vitex Agnus-castus* by the Serotonergic System

**Published:** 2012

**Authors:** Parichehr Yaghmaei, Shahrbanoo Oryan, Laleh Fatehi Gharehlar, Ali-Akbar Salari, Jalal Solati

**Affiliations:** 1 Department of Animal Biology, Science and Research Campus, Islamic Azad University, Tehran, Iran; 2 *Tarbiat Moalem University of Tehran, Tehran, Iran*; 3 *Department of Biology, North Tehran Branch, Islamic Azad University, Tehran, Iran*; 4 *Department of Biology, Karaj Branch, Islamic Azad University, Karaj, Iran*

**Keywords:** Anxiety, Rat, Serotonergic system, Vitex agnus-castus

## Abstract

**Objective(s):**

There is well documented evidence for the increase in widespread use of complementary and alternative medicine in the treatment of physical and psychiatric symptoms and disorders within the populations. In the present study, we investigated the influence of *Vitex agnus-castus* (vitex) on anxiety-like behaviors of rats.

**Materials and Methods:**

Elevated plus maze which is one of the methods used for testing anxiety is used in our present study. Rats were orally administrated with vitex for two week. The anxiety test was carried out after two weeks of oral administration of vitex. For evaluating interaction of vitex and serotonergic systems, rats were anaesthetized with ketamine and special cannulas were inserted stereotaxically into the third ventricle (TV) of brain. After 1 week recovery, the effects of serotonegic agents on anxiety were studied.

**Results:**

Oral administration of vitex (100, 200, 300 mg/kg) for two weeks induced an anxiogenic-like effect which was shown through specific decreases in the percentages of open arm time (OAT %) and open arm entries (OAE %). *Intra-TV* infusion of 5HT_1A_ receptor agonist, 8-OH-DPAT (5, 10 and 25 ng/rat) increased OAT% and OAE%, indicating anxiolytic–like behavior. However, injection of 5HT_1A_ receptor antagonist NAN190 (0.25, 0.5 and 1 µg/rat) produced anxiogenic-like behavior. The most effective dose of 8-OH-DPAT (10 ng/rat), when co-administered with vitex (100, 200, 300 mg/kg), attenuated the anxiogenic-like effects of vitex significantly. Injection of the less effective dose of NAN190 (0.5 µg/rat), in combination with vitex (100, 200, 300 mg/kg), potentiate anxiogenic effects of vitex.

**Conclusions:**

These results illustrate that 5HT_1A_ receptor is involved in the anxiogenic effects of vitex.

## Introduction

The use of herbal medicine is increasing worldwide. It has been estimated that 80% of the world's population (four billion people) use some form of herbal medicine as part of their primary health care (1, 2). However, herbal medicines are not always the harmless nostrums that many patients and even some physicians think, conversely, they may contribute to hepatic injury, mental disorder and cardiovascular morbidity and mortality (-). A herb containing a wide variety of (mostly unknown) substances may well include some with unwanted effects; a treatment method using a mechanical procedure may lead to injuries; and a healer without adequate education may be a cause of increased diagnostic and treatment faults. Over recent years, numerous case reports and some controlled studies have been published on adverse effects of complementary and herbal medicine (3). For over 2500 years *Vitex agnus-castus (*vitex; chaste tree) has been used for gynecological conditions since the days of Hippocrates. Nowadays ethanolic extracts from the fruit are used world widely for the treatment of mastopathy, premenstrual syndrome and luteal insufficiency-complaints caused by a mild or a latent hyperprolactinemia (6, 7). The mechanisms that involved the effects of vitex are proposed to be due to the brain neurotransmitter systems such as dopaminergic, serotonergic and estrogenic in nature (-). Anxiety disorders are the most common type of psychiatric disorders, with an incidence of 18.1% and a lifetime prevalence of 28.8% (10). In the United States the economic costs of anxiety disorders is an estimated 42.3 billion dollars each year and the lifetime medical costs for an individual with an anxiety disorder is almost $6,475, and even more among patients with disorders such as panic, generalized anxiety, and post-traumatic stress (11). These days complementary and alternative therapies are more common among people with psychiatric problems than the rest of the population because anxiety, depression and insomnia are among the most commonly reported reasons for the use of complementary and alternative therapies in community surveys (2, 12). Today, various herbal remedies around the world, are used for the treatment of anxiety and there is an enormous interest in search for medicinal plants which are effective in treatment of anxiety (7, 13). Previous studies showed that vitex interacts with neurotransmitter and neural system, such as dopaminergic, serotonergic which are involved in modulation of anxiety (-). Therefore, the present study aimed to evaluate the effects of vitex on anxiety-like behaviors of rats and possible interaction between vitex and serotonergic system in the modulation of anxiety-like behaviors.

## Materials and Methods

***Animals***

Male Wistar rats from Pasteur Institute of Iran (Tehran) were used with the weight of 180–230 g at the time of surgery. Four animals were housed in each cage, in a room with a 12:12 hr light / dark cycle (lights on 07:00 hr) and controlled temperature (23 ± 1 °C), and they had access to food and water *ad libitum* and were allowed to adapt to the laboratory conditions for at least one week before surgery. The rats were handled about 3 min each day prior to behavioral testing. All the experiments were performed between 12:00 and 15:00 hr, and each rat was tested only once. Seven animals were used in each experiment. Animal studies were conducted according to the guidelines of the NIH guidelines for the care and use of animals in research.

***Stereotaxic surgery and microinjections***

The rats were anesthetized intraperitoneally with ketamine hydrochloride (50 mg/kg) and xylazine (4 mg/kg) and placed in a Stoelting stereotaxic instrument; a stainless steel guide cannula (22-gauge) was implanted in the *third ventricular* *(TV)* regions according to Paxinos and Watson (17). Stereotaxic coordinates for the *third ventricular* regions were: − 3.8 mm posterior to bregma, ± 2 mm lateral to the midline and − 3 mm ventral of the dorsal surface of the skull. The cannula was fixed to the skull with acrylic dental cement. The animals were allowed 5 days before the test to recover from surgery. The *third ventricular* was infused by means of an internal cannula (27-gauge), terminating 1 mm below the tip of the guides, connected by polyethylene tubing to a 1-μl Hamilton syringe. On each side 0.5 μl solution was injected (1 μl/rat) over a 60 sec period. The inner cannula was left in place for an additional 60 sec to allow diffusion of the solution and to reduce the possibility of reflux. Intra-*third ventricular* injections were made 5 min before testing. 

***Elevated plus-maze***

This wooden, plus-shaped apparatus was elevated to the height of 50 cm above the floor. This apparatus was composed of two 50×10 cm open arms, and two 50×10×50 cm enclosed arms, each with an open roof. The maze was placed in the center of a quiet and dimly lit room. The rats' behavior was directly observed using a mirror, suspended at an angle above the maze. Behavioral data were collected by a “blind” observer who quietly sat 1 m behind one of the closed arms of the maze, using a time recorder. Five min following their respective drug treatment, rats were placed individually in the center of the plus-maze, facing one of the closed arms. The observer measured (1) time spent in the open arms, (2) time spent in the closed arms, (3) number of entries into the open arms, and (4) number of entries into the closed arms during the 5-min test period. An entry was defined as all four paws in the arm. The maze was cleaned with distilled water after each rat was tested. For the purpose of analysis (-), open-arm activity was quantified as the amount of time that the rat spent in the open arms in comparison with the total amount of time spent in any arm (open/total×100), and the number of entries into the open arms was quantified in comparison with the total number of entries into any arm (open/total×100). The total number of open arms entered, as well as the total number of closed arms entered, was used as indexes of general locomotor activity (18, -). 

***Drugs***

The drugs used in the present study were *Vitex agnus-castus* (Poorsina, Co., Iran), (±)-8-hydroxy-2-(di-n-propyl-amino) tetralin hydrobromide (8-OH-DPAT; Sigma, USA), NAN190 hydrobromide (Sigma Chemical Co., USA), 8-OH-DPAT and NAN190 were dissolved in sterile 0.9% saline. Control animals received saline. Drug solutions were freshly prepared before administration.

For evaluation of possible interaction between vitex and 5HT_1A_ receptor agonist and antagonist, rats were treated with vitex for 14 days; in the day 15 agonist or antagonist received intra* third ventricular* injection. 5 min after intra* third ventricular* injection each rat moved to EPM for behavioral analysis. All of the injections and behavioral assessments were done between 16 - 18 p.m. 

***Drug treatments***

*Experiment 1: The effects of* *vitex* *on anxiety-like* *behavior (unpaired group)*

For evaluating the effects of vitex on anxiety, rats were divided to four groups. Three groups were orally (gavages) administrated with vitex (100, 200, 300 mg/kg), and one of the above four groups received saline (control group) for two weeks.

*Experiment 2: The* *effects of* *5HT*_1A_ *receptor agonist* *and antagonist on anxiety-like behavior* *(unpaired group)*

Three groups of rats received intra* third ventricular* injection of selective 5HT_1A_ receptor agonist 8-OH-DPAT (5, 10 and 25 ng/rat) and three other groups received 5HT_1A_ antagonist NAN190 (0.25, 0.5 and 1 µg/rat). These rats were thereafter compared with a saline control group.

*Experiment 3:* *The effects of* *vitex* *by itself or with* *8-OH-DPAT* *on anxiety-like* *behavior* (paired group) 

First four groups of rats were orally treated with saline or vitex (100, 200, 300 mg/kg) for two weeks. After two weeks oral administration of saline and vitex all groups received saline (1 μl/rat, intra *third ventricular*).

Four other groups of rats received saline or vitex (100, 200, 300 mg/kg) orally for two weeks. After two weeks oral treatment of saline and vitex, al1 treated rats were injected with 8-OH-DPAT (10 ng/rat, intra *third ventricular*).

*Experiment 4:* *The effects of* *vitex* *by itself or with* *NAN190* *on anxiety-like* *behavior (paired group) *First four groups of rats orally received saline or vitex (100, 200, 300 mg/kg) for two weeks. After two weeks oral administration of saline and vitex, all groups received saline (1 μl/rat, intra *third ventricular*). 

Four other groups of rats were orally treated with saline or vitex (100, 200, 300 mg/kg) for two weeks. After two weeks of oral treatment, saline and vitex, treated rats were injected with the most effective dose of NAN190 (0.5 µg/rat, intra *third ventricular*).

***Statistical analysis***

Since the data displayed normal distribution and homogeneity of variance, one-way ANOVA was used for comparing the effects of different doses of drugs with vehicle. Two-way ANOVA was used for evaluation of interactions between drugs. In the case of significant differences, the *post hoc* analysis (least significant difference, LSD) was performed to assess specific group comparisons. Differences with *P*< 0.05 between experimental groups at each point were considered statistically significant.

## Results

***The effects of*** ***vitex*** ***on anxiety-like*** ***behavior***

**Figure 1:**
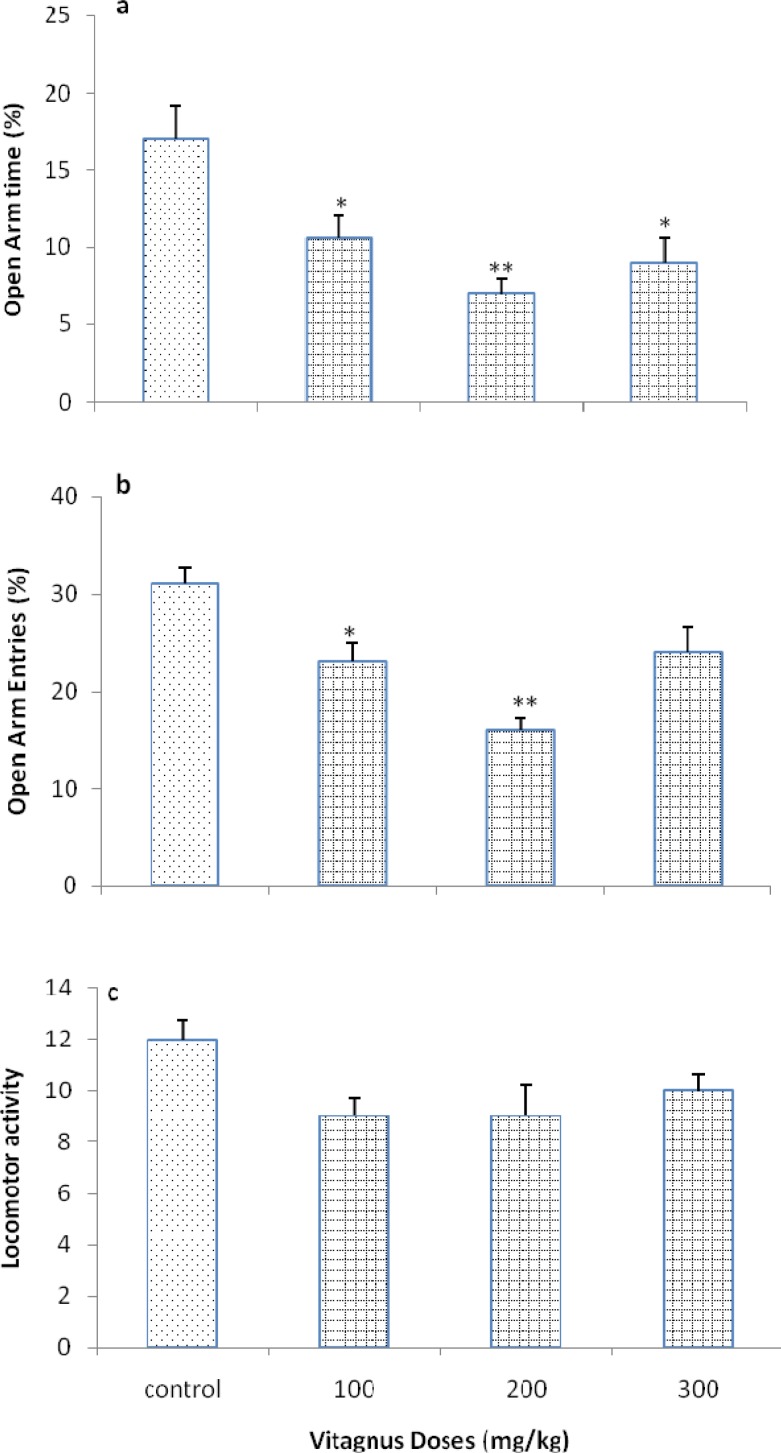
Effects of oral administration of vitex in the EPM. Rats were treated with either saline (1 ml/rat) or with vitex (100, 200, and 300 mg/rat). Each bar is mean ± SEM. *N* = 7. *P < 0.05 and **P < 0. 01, when compared to the saline treated rats (One-way ONOVA)

Figure 1 shows the effect of continues oral treatment of vitex (100, 200, 300 mg/kg) on anxiety-like behaviors of rats. The one-way ANOVA revealed that vitex decreased OAT% [*F* (3, 24)= 10.345, *P*< 0.05], OAE% [*F* (3, 24) = 9.112, *P* < 0.05] at the doses of 100, 200, and 3000 mg/kg, indicating the induction of anxiogenic-like response by vitex. No changes were observed in the locomotor activity [*F* (3, 24) = 2.292, *P* > 0.05].

***The effects of*** ***5HT***_1A_ ***receptor agonist and antagonist on anxiety-like behavior***

**Figure 2:**
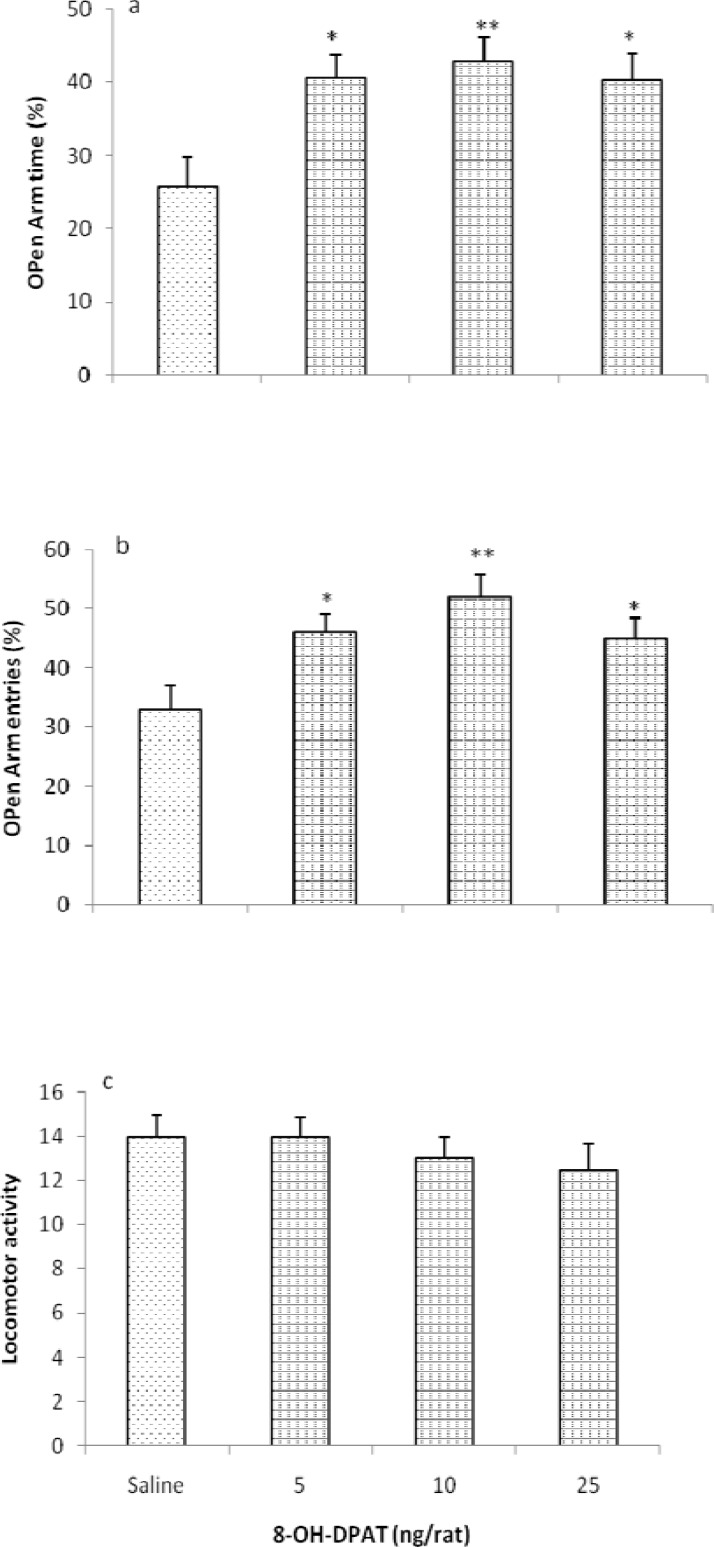
Effects of intra-third ventricle injection of 8-oh-DPAT in the EPM. Rats were treated with either saline (1 μl/rat) or with 8-oh-DPAT (5, 10 and 25 ng/rat). Each bar is mean ± SEM. *N* = 7. *P < 0.05 and **P < 0.01, when compared to the saline treated rats (One-way ONOVA)

Figure 2 shows the effect of intra-TV injection 5HT_1A_ receptor agonist, 8-OH-DPAT (5, 10 and 25 ng/rat) in the EPM in rats. Our results shows that, 8-OH-DPAT increased OAT% [*F* (3, 24) = 12.354, *P* < 0.05] and OAE% [*F* (3, 24) =10.646, *P* < 0.05], indicating the induction of anxiolytic-like response by 8-OH-DPAT. No changes were observed in the locomotor activity [*F* (3, 24) = 1.121, *P* > 0.05].

**Figure 3:**
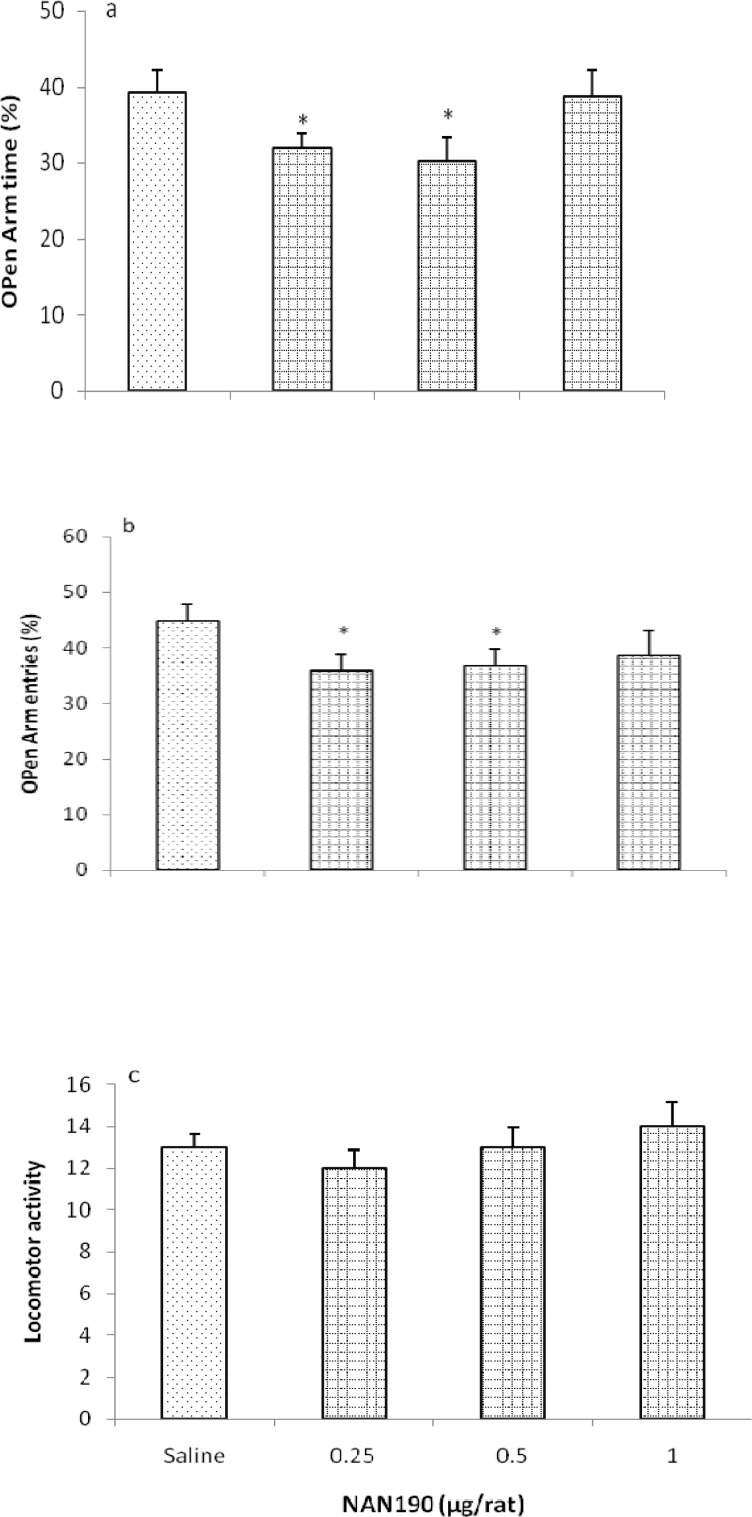
Effects of intra-third ventricle injection of NAN190 in the EPM. Rats were treated with either saline (1 μl/rat) or with NAN190 (0.25, 0.5 and 1µg/rat intra-TV). Each bar is mean ± SEM. *N* = 7. *P < 0.05, when compared to the saline treated rats (One-way ONOVA)

However, the rats infused with NAN190 (0.25, 0.5, and 1 μg/rat, intra-TV) showed significant decrease in OAT% [*F* (3, 24) = 4.484, *P* < 0.05] at dose of 0.5 μg/rat and OAE% [*F* (3, 24) = 5.564, *P* < 0.05] at the doses 0.25 and 0.5 μg/rat in comparison with the saline-infused controls. No changes were observed in the locomotor activity [*F* (3, 24) = 2.014, *P* > 0.05] (Figure 3).

***The effects of vitex by itself or with 8-OH-DPAT on anxiety-like behavior***

**Figure 4:**
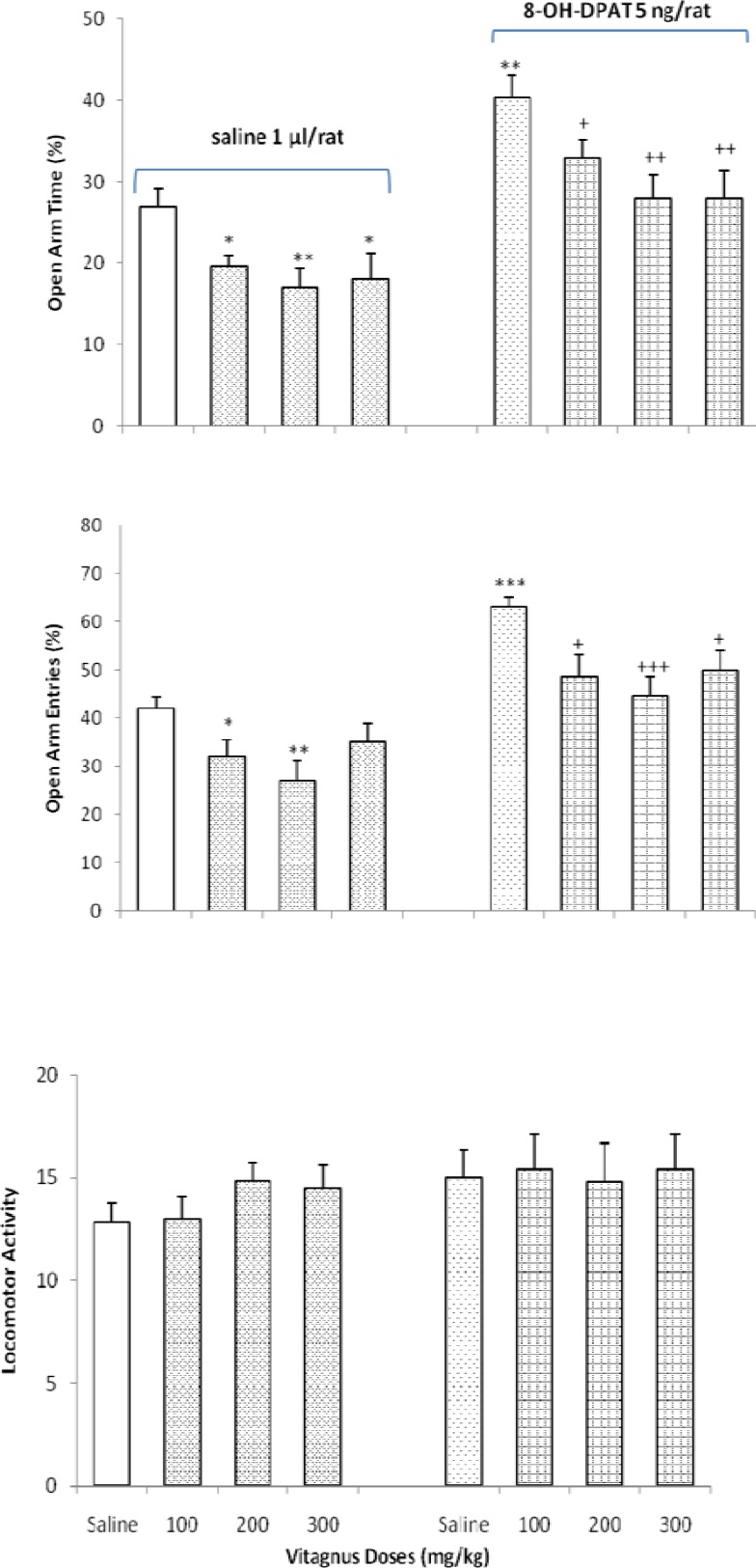
Effects of vitex administration in the absence or presence of 8-oh-DPAT on anxiety-like behavior in the EPM. *P < 0.05, **P < 0.001 and ***P<0.001, compared saline/saline control group. +*P* < 0.05, ++*P* < 0.01 and +++*P* < 0.001 when compared to saline/8-oh-DPAT control group. (Two-way ONOVA)

The effects of intra-TV injections of the 5HT_1A_ receptor agonist, 8-OH-DPAT (5 µg/rat) on the response induced by vitex (100, 200, 300 mg/kg) is shown in Figure 4. The two-way ANOVA indicated that the combination of vitex (Factor A) with 8-OH-DPAT (Factor B) showed an interactions for OAT% [Factor A; *F* (3,48) = 7.562, *P* < 0. 01, Factor B; *F* (1,48) = 5.252, *P* < 0.05, Factor (A × B); *F* (3,48) = 4.133, *P* < 0.05], for OAE% [Factor A; *F* (1,48) = 6.956, *P* < 0. 01, Factor B; *F* (1,48) = 6.624, *P* < 0.01, Factor (A × B); *F* (3,48) = 3.952, *P* < 0.05], and for locomotor activity [Factor A; *F* (1,48) = 2.058, *P*> 0.05, Factor B; *F* (1,48) = 2.142, *P* > 0.05, Factor (A × B); *F* (3,48) = 1.131, *P*> 0.05]. 

**Figure 5:**
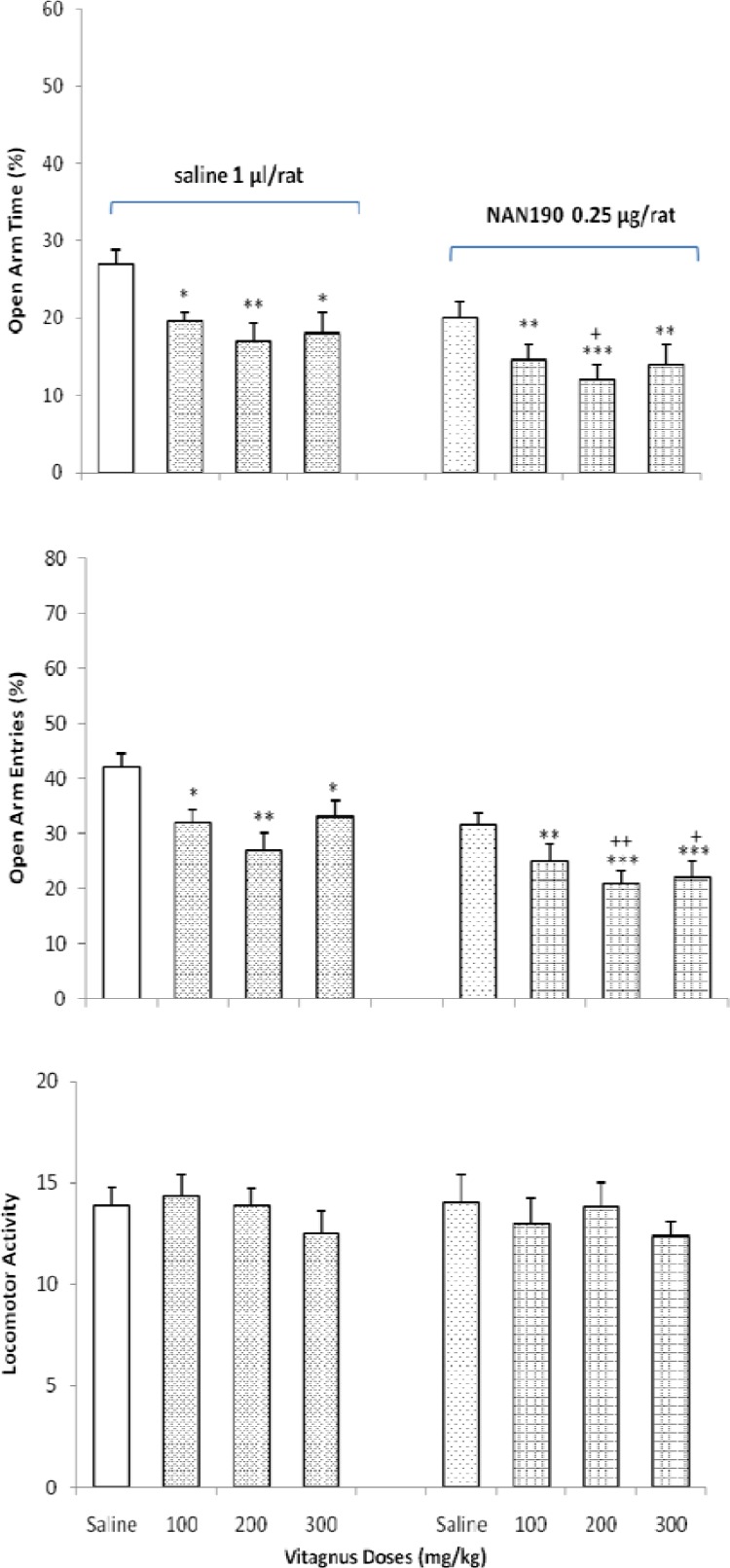
Effects of vitex administration in the absence or presence of NAN190 on anxiety-like behavior in the EPM. *P < 0.05, **P < 0.001 and ***P<0.001, compared saline/saline control group. +*P* < 0.05and ++*P* < 0.01 when compared to saline/ NAN190 control group. (Two-way ONOVA).

***The effects of vitex by itself or with NAN190 on anxiety-like behavior***

Figure 5 shows the effects of oral administration of vitex in the presence or absence of receptor antagonist, NAN190 (0.5 μg/rat) on anxiety-like behavior. Two-way ANOVA showed an interactions between the effects of vitex (Factor A) and NAN190 (Factor B) on OAT% [Factor A; *F* (4,60) = 2.111, *P* < 0.05, Factor B; *F* (1,60) = 12.226, *P* < 0.001, Factor (A × B); *F* (4,60) = 4.215, *P* < 0.05], OAE% [Factor A; *F* (4,60) = 4.142, *P* < 0.001, Factor B; *F* (1,60) = 16 .161, *P* < 0.001, Factor (A × B); *F* (4,60) = 6.221, *P* < 0.01], and locomotor activity [Factor A; *F* (4,60) = 0.325, *P* > 0.05, Factor B; *F* (1,60) = 2.126, *P* > 0.05, Factor (A × B); *F* (4,60) = 1.124, *P* > 0.05].

## Discussion

There is a well documented evidence for the increase in the widespread use of complementary and alternative medicine in the treatment of physical and psychiatric symptoms and disorders within populations (-). Our experiments indicated that 2 week oral administration of the vitex decreased both OAT% and OAE%, without locomotor impairment in the EPM which indicated the induction of anxiogenic-like response by vitex. 

Other parts of this experiments revealed that the intra *third ventricular* injection of 5HT_1A_ receptor agonist; 8-OH-DPAT increased both OAT% and OAE%, without locomotor impairment in the EPM which indicated the induction of anxiolytic-like response by 8-OH-DPAT. Furthermore, intra *third ventricular* administration of 5HT_1A_ receptor antagonist, NAN190 produced anxiogenic-like effects in the EPM, without any significant effects on locomotor activity.

The results of the present study also showed that the administration of 8-OH-DPAT after two week treatment with vitex, attenuate anxiogenic-like effects of vitex. However, the administration of NAN190 after continues treatment of vitex; potentiate anxiogenic-like effects of vitex, indicating an interaction between vitex and 5HT_1A_-serotonergic system. 

The brain serotonin system plays an important role in the neural processing of anxiety. The involvement of the main inhibitory serotonergic receptor, the serotonin-1A (5-HT_1A_) subtype, in dysfunctional forms of anxiety has been supported by findings from a wide range of preclinical research and clinical trials, including treatment studies, genetic research, and neuroimaging data (30, 31). Partial agonists at 5-HT_1A_ receptor, such as buspirone, are in clinical use as anxiolytics (32, 33), and 5-HT_1A_ antagonists are reported to accelerate the therapeutic effects of antidepressant medications and increase anxiety (-). These agonists and antagonists play their effects via pre-synaptic or postsynaptic 5-HT_1A_ receptors in the different parts of the brain (30, 37). 

5-HT_1A_ receptors are located on serotonergic neuronal cell bodies and dendrites are the predominant somatodendritic autoreceptors of these neurons; their activation suppresses serotonergic neuronal activity (35, 38). In addition, postsynaptic 5-HT_1A_ receptors are expressed in numerous serotonergic projection sites such as the cerebral cortex, septalnuclei, hippocampus, and amygdale (35, 39). All of these areas are involved in the modulation of anxiety (-).

## Conclusions

Our results demonstrated that continues oral treatment with vitex produce an anxiogenic-like effect in rats. Intra *third ventricular* administration of serotonin 5HT_1A_ receptor agonist, 8-OH-DPAT, decreases anxiety-like in rats, while administration of NAN190 potentiate anxigenic-like effects of vitex. The data possibly indicated that the anxiogenic-like effects of vitex were at least partially modulated by 5HT_1A_ receptors. 

## References

[1] Bennett J, Brown C (2000). Use of herbal remedies by patients in a health maintenance organization. J Am Pharm Assoc.

[2] Eisenberg D, Davis R, Ettner S, Appel S, Wilkey S, Van RompayM (1998). Trends in alternative medicine use in the United States, 1990-1997: results of a follow-up national survey. JAMA.

[3] Niggemann B, Grüber C (2003). Side effects of complementary and alternative medicine. Allergy.

[4] Wong A, Smith M, Boon H (1998). Herbal remedies in psychiatric practice. Arch Gen Psychiatry.

[5] MacGregor F, Abernethy V, Dahabra S, Cobden I, Hayes P (1989). Hepatotoxicity of herbal remedies. Brit Med J.

[6] Cossuta D, Simándi B, Vági E, Hohmann J, Prechl A, Lemberkovics É (2008). Supercritical fluid extraction of Vitex agnus- castus fruit. J Supercrit Fluids.

[7] Wuttke W, Jarry H, Christoffel V, Spengler B, Seidlova-Wuttke D (2003). Chaste tree (Vitex agnus-castus)-pharmacology and clinical indications. Phytomedicine.

[8] Webster D, Lu J, Chen S, Farnsworth N, Wang Z (2006). Activation of the [mu]-opiate receptor by Vitex agnus-castus methanol extracts: Implication for its use in PMS. J ethnopharmacol.

[9] Van DieM, Burger H, Bone K, Cohen M, Teede H (2009). Hypericum perforatum with Vitex agnus-castus in menopausal symptoms: a randomized, controlled trial. Menopause.

[10] Kessler R, Chiu W, Demler O, Walters E (2005). Prevalence, severity, and comorbidity of 12-month DSM-IV disorders in the national comorbidity survey replication. Arch Gen Psychiatry.

[11] Deacon B, Lickel J, Abramowitz JS (2008). Medical utilization across the anxiety disorders. J Anxiety Disorders.

[12] Kessler R, Soukup J, Davis R, Foster D, Wilkey S, Van RompayM (2001). The use of complementary and alternative therapies to treat anxiety and depression in the United States. Am J Psychiatry.

[13] Ernst E (2006). Herbal remedies for anxiety-a systematic review of controlled clinical trials. Phytomedicine.

[14] Zarrindast M, Babapoor-Farrokhran S, Rezayof A (2008). Involvement of opioidergic system of the ventral hippocampus, the nucleus accumbens or the central amygdala in anxiety-related behavior. Life sciences.

[15] Lesch K, Bengel D, Heils A, Sabol S, Greenberg B, Petri S (1996). Association of anxiety-related traits with a polymorphism in the serotonin transporter gene regulatory region. Science.

[16] Simon P, Dupuis R, Costentin J (1994). Thigmotaxis as an index of anxiety in mice Influence of dopaminergic transmissions. Behav Brain Res.

[17] Paxinos G (2007). The Rat Brain in Stereotaxic Coordinates.

[18] Degroot A, Kashluba S, Treit D (2001). Septal GABAergic and hippocampal cholinergic systems modulate anxiety in the plus-maze and shock-probe tests. Pharmacol Biochem Behav.

[19] Pellow S, Chopin P, File SE, Briley M (1985). Validation of open:closed arm entries in an elevated plus-maze as a measure of anxiety in the rat. J Neurosci Methods.

[20] Pellow S (1986). Anxiolytic and anxiogenic drug effects in a novel test of anxiety: are exploratory models of anxiety in rodents valid. Methods Find Exp Clin Pharmacol.

[21] Rodgers RJ, Johnson NJ (1995). Factor analysis of spatiotemporal and ethological measures in the murine elevated plus-maze test of anxiety. Pharmacol Biochem Behav.

[22] Zarrindast MR, Solati J, Oryan S, Parivar K (2008). Effect of intra-amygdala injection of nicotine and GABA receptor agents on anxiety-like behaviour in rats. Pharmacology.

[23] Thomas K, Coleman P (2004). Use of complementary or alternative medicine in a general population in Great Britain Results from the National Omnibus survey. J Public Health.

[24] Lengacher C, Bennett M, Kip K, Keller R, LaVance M, Smith L (2002). Frequency of use of complementary and alternative medicine in women with breast cancer. Onc Nurs Society.

[25] van derWattG, Laugharne J, Janca A (2008). Complementary and alternative medicine in the treatment of anxiety and depression. Curr Opin Psychiatry.

[26] Barnes P, Powell-Griner E, McFann K, Nahin R (2004). Complementary and alternative medicine use among adults.

[27] Angell M, Kassirer J (1998). Alternative medicine--the risks of untested and unregulated remedies. N Engl J Med.

[28] Kozyrskyj A (1997). Herbal products in Canada. How safe are they. Can Fam Physician.

[29] Ernst E (2007). Herbal medicines: balancing bene ts and risks. Dietary supplements and health.

[30] Akimova E, Lanzenberger R, Kasper S (2009). The serotonin-1A receptor in anxiety disorders. Biol Psychiatry.

[31] Lanzenberger R, Mitterhauser M, Spindelegger C, Wadsak W, Klein N, Mien L (2007). Reduced serotonin-1A receptor binding in social anxiety disorder. Biol Psychiatry.

[32] Goldberg H, Finnerty R (1979). The comparative efficacy of buspirone and diazepam in the treatment of anxiety. Am J Psychiatry.

[33] Gammans R, Stringfellow J, Hvizdos A, Seidehamel R, Cohn J, Wilcox C (1992). Use of buspirone in patients with generalized anxiety disorder and coexisting depressive symptoms. Neuropsychobiology.

[34] Artigas F, Romero L, de MontignyC, Blier P (1996). Acceleration of the effect of selected antidepressant drugs in major depression by 5-HT1A antagonists. Trends Neurosci.

[35] Heisler L, Chu H, Brennan T, Danao J, Bajwa P, Parsons L (1998). Elevated anxiety and antidepressant-like responses in serotonin 5-HT1A receptor mutant mice. Proc Natl Acad Sci.

[36] Belcheva I, Belcheva S, Petkov V, Hadjiivanova C, Petkov V (1997). Behavorial responses to the 5-HT1A receptor antagonist NAN190 injected into rat CA1 hippocampal area. Gen Pharmacol.

[37] File S, Gonzalez L, Andrews N (1996). Comparative study of pre-and postsynaptic 5-HT1A receptor modulation of anxiety in two ethological animal tests. J Neurosci.

[38] Kennett G, Marcou M, Dourish C, Curzon G (1987). Single administration of 5-HT1A agonists decreases 5-HT1A presynaptic, but not postsynaptic receptor-mediated responses: relationship to antidepressant-like action. Eur J Pharmacol.

[39] Jolas T, Schreiber R, Laporte A, Chastanet M, De VryJ, Glaser T (1995). Are postsynaptic 5-HT1A receptors involved in the anxiolytic effects of 5-HT1A receptor agonists and in their inhibitory effects on the firing of serotonergic neurons in the rat. J Pharmacol Exp Ther.

[40] Chua P, Krams M, Toni I, Passingham R, Dolan R (1999). A functional anatomy of anticipatory anxiety. NeuroImage.

[41] Kazer J, Sharkey A (1999). The septo-hippocampal system and anxiety. Artificial Neural Networks.

[42] Davis M (1992). The role of the amygdala in fear and anxiety. Ann Rev Neurosci.

